# Supplementary material for: Analysis of the spatial association of geographical detector-based landslides and environmental factors in the southeastern Tibetan Plateau, China
Source: PLoS One. 2021 May 20;16(5):e0251776. doi: 10.1371/journal.pone.0251776 (PMC8136732; doi:10.1371/journal.pone.0251776)
Supplement: Attachment — Submitted filename: Reviewer note.pdf [file pone.0251776.s001.pdf]

The main objective of manuscript is to explore the spatial association between the landslides and the environmental factors in the southeastern part of Tibet Plateau. The subject of study is interesting and article is generally well structured. However, I would like to recommend the authors to improve the paper considering the following comments.

1-Many studies have been conducted to analysis influencing factors on landslides as a typical disaster in china. Author(s) also mentioned some of them in introduction section. The concluding remark of literature review in Introduction is "none of these studies would highlight the dominant factors or the interaction between the factors, and the existing landslides investigation is incomplete". However, the new findings of this study should be clear and must compare to other studies in discussion and authors should emphasize on their new finding. Manuscript needs more about innovation and validation.

2-Geographical detector tool has applied to detect the dominant factors; this statistical method includes four parts which are risk detector, factor detector, ecological detector, and interaction detector. Author(s) are recommended to provide some information if other sophisticated methods are available to do such analysis and provide evidence for the significance and preference of their methodology based on other studies.

3-It is clear that different factors and multi-factor interaction can influence landslides, but the challenge is to find what those interactions are, how much influence of which factor, how the changes on those factors influence the changes of landslides and what can be the reasons of them. It is required to put more scientific interpretation of results and I suggest the authors to improve the discussion.

4- The quality of the figures is very poor. Please replace all with good ones. The figure1 were not defined in terms of A, B, C, D, F. Landslide inventory map can provide with good quality and the location of study area can be presented.

5- Grammar and English writing need to improve.

6-keywords should insert after abstract and before introduction.

7-For the link: http://www. GeoDetector.org, Please check as it does not exist, do you mean http://www.geodetector.cn/ ?

8- You May reconsider about your title as there is much similarity with below reference:

Yue, X. L., Wu, S. H., Huang, M., Gao, J. B., Yin, Y. H., Feng, A. Q., & Gu, X. P. (2018). Spatial association between landslides and environmental factors over Guizhou Karst Plateau, China. *Journal of Mountain Science*, *15*(9), 1987-2000. https://link.springer.com/article/10.1007%2Fs11629-018-4909-2
