# Supplementary material for: Analysis of the spatial association of geographical detector-based landslides and environmental factors in the southeastern Tibetan Plateau, China
Source: PLoS One. 2021 May 20;16(5):e0251776. doi: 10.1371/journal.pone.0251776 (PMC8136732; doi:10.1371/journal.pone.0251776)
Supplement: Attachment — Submitted filename: Comments to author1.docx [file pone.0251776.s002.docx]

**Comments to authors**

The authors have addressed all the comments and observations. However, I still have some few observations.

There are still some figures that the texts are not clear. Authors should make these texts legible.
