# Supplementary material for: Analysis of the spatial association of geographical detector-based landslides and environmental factors in the southeastern Tibetan Plateau, China
Source: PLoS One. 2021 May 20;16(5):e0251776. doi: 10.1371/journal.pone.0251776 (PMC8136732; doi:10.1371/journal.pone.0251776)
Supplement: Attachment — Submitted filename: Response to Reviewers.docx [file pone.0251776.s003.docx]

**Manuscript PONE-D-20-11323**

**Response to Reviewers**

Dear academic editor:

Thank you for giving us the opportunity to summit a revised draft of the manuscript “Spatial association analysis between landslides and environmental factors based on geographical detector in southeastern of Tibet Plateau, China” for publication in the PLOS ONE. We appreciate the valuable time and effort that you and reviewers dedicated to providing feedback on our first revised manuscript. After careful consideration and revision, we give our responds to each point raised by you and reviewers. The point-by-point response to and requirements and comments in blue, please see below.

**Journal Requirements:**

1) Please review your reference list to ensure that it is complete and correct. If you have cited papers that have been retracted, please include the rationale for doing so in the manuscript text, or remove these references and replace them with relevant current references. Any changes to the reference list should be mentioned in the rebuttal letter that accompanies your revised manuscript. If you need to cite a retracted article, indicate the article’s retracted status in the References list and also include a citation and full reference for the retraction notice.

**Author response**: Thank you for pointing this out! According to this requirement, we reviewed each reference carefully in the reference list of our manuscript to ensure that it is complete and correct. There are two references with incomplete or incorrect information, which are reference [47] and reference [62], and we have changed them in 'Revised Manuscript with Track Changes' and 'Manuscript'. Then, after detail reviewed, all of our cited paper are not retracted and could check their information in the website of publishers or journals.

2) Please state in your Methods section whether any permits were obtained for the field work, or if no field permits were obtained, a short statement as to why not.

**Author response**: The Qinghai-Tibet Plateau has unique topographical landscape and scientific research significance, and the Chinese Academy of Sciences carries out various scientific research investigations here every year. Our Field work obtained the permission from our school and local governments. We have state that permit in “Manuscript” and “Revised Manuscript with Track Changes” from Line200 to Line 204.

**Comments to author**:

1. The authors have addressed all the comments and observations. However, I still have some few observations. There are still some figures that the texts are not clear. Authors should make these texts legible.

**Author response**: Thank you for point this out, we check all the figures in our manuscript in Electronic and printed document. Then we changed the sizes and colors to ensure their legible in Figure1, Figure2, Figure3 and Figure8. And all the figures will be upload to the website, if you have any further questions, please contact us again.

Thank you again!

Best Wishes!

Weijie
